# In vitro effect of glucocorticoids on nasal polyps

**DOI:** 10.1590/S1808-86942011000500012

**Published:** 2015-10-22

**Authors:** Fabiana Valera, María S. Brassesco, Angel M. Castro-Gamero, Maria A. Cortez, Rosane G.P. Queiroz, Luiz G. Tone, Wilma T. Anselmo-Lima

**Affiliations:** 1 PhD, MD (professora doutora da FMRP - USP); 2 PhD (jovem pesquisadora associada ao Laboratório de Pediatria da FMRP-USP); 3 Geneticist (mestrando no Laboratório de Pediatria da FMRP-USP); 4 Geneticist (doutora pelo Laboratório de Pediatria da FMRP); 5 PhD (coordenadora do Laboratório de Pediatria da FMRP); 6 PhD, MD (professor titular da FMRP); 7 PhD, MD (professora associada da FMRP)

**Keywords:** adrenal cortex hormones, nasal polyps, NF-kappa B, sinusitis

## Abstract

**Abstract:**

Glucocorticoids are considered the main treatment option for nasal polyps, but their effect is only recently being understood.

**Aim:**

To evaluate whether fluticasone propionate (FP) inhibits the inflammatory process induced by TNF-alpha *in vitro*, and to assess if NF-kappaB is associated to this inhibition.

**Study Design:**

Experimental *in vitro* study.

**Materials and Methods:**

Nasal polyp fibroblasts were cultured during 24 hours. Three different concentrations of FP (1, 10 and 100 nM, added to TNF-alpha) were compared to negative (without additive) and positive (TNF-alpha) controls. Gene expression (RTQ-PCR) and protein concentration (ELISA) of VCAM-1, ICAM-1, eotaxin and RANTES were measured, as well as the nuclear translocation of NF-kappaB.

**Results:**

TNF-alpha significantly increased protein concentration and RNA expression of all the studied molecules, as well as the nuclear translocation of NF-kappaB, when compared to the negative control. FP decreased these parameters in a dose-dependent manner, statistically different from positive control up to 100nM.

**Conclusions:**

FP extensively inhibited inflammatory recruiters, at both protein and RNA levels, confirming the ability of glucocorticoids to modulate the inflammatory process in nasal polyps. This inhibition was associated to decreased NF-kappaB nuclear translocation, demonstrating that this is an important mechanism of glucocorticoids action for nasal polyps.

## INTRODUCTION

Nasal polyps (NP) is an inflammatory disease, which primarily affects the sinonasal mucosa^1^,^2^. Inflammation is generally induced by pro-inflammatory cytokines, such as TNF-α (tumor necrosis factor-a) and IL-1ß (interleukin-1ß) and mediated by transcriptional factors (TF), such as NF-κß (nuclear factor-κß) and AP-1 (activator protein-1)^3^. Initially, TFs translocate into the nucleus and induce the expression of pro-inflammatory molecules in nasal structural cells (fibroblasts, epithelial and endothelial cells)^3^, ^4^, ^5^, ^6^, ^7^, which in turn produce chemokines and adhesion molecules (including eotaxin), RANTES (regulated upon activation normally Texpressed and secreted), ICAM-1 (intercellular adhesion molecule-1), VCAM-1 (vascular cell adhesion molecule-1), E-selectin and P-selectin), that will induce the migration of inflammatory cells (such as T lymphocytes, eosinophils, mast cells, and neutrophils) towards the target^7^, ^8^, ^9^. Once on the nasal mucosa, the circulating cells will promote tissue damage^8^,^10^, ^11^, ^12^. Bachert et al.^2^ stated that deregulation of chemokines and adhesion molecules production might be important in promoting the local chemotaxis of eosinophils.

According to the European Position Paper on Rhinosinusitis and Nasal Polyps 200^7^,^11^ and other researchers^12^, ^13^, ^14^, ^15^, topical glucocorticoids (GC) are the cornerstone for treating NP. However, the success rate of topical GC ranges from 60.9 to 80%^12^,^13^,^16^. The anti-inflammatory property of GCs occurs due to their binding to the glucocorticoid receptor (GR); the GC-GR complex inhibits other TF, such as NF-κß, a phenomenon known as transrepression^4^,^17^, ^18^, ^19^. The repression of NF-κß will finally inhibit the expression of some cytokines as TNF-α, IL-1, IL-8 (interleukin-8) and ICAM-1,^17^. Transrepression between GCs and TFs is reciprocal, and NF-κß^17^ can also repress glucocorticoid receptors; which could, at least in part, induce GC resistance in some patients.

Recently^20^, our group has observed that *in-vivo* budesonide treatment leads to a significant improvement in symptoms and endoscopic reduction of nasal polyps. Despite this significant clinical improvement, only one patient reached complete remission. Moreover, we observed that patients with an unfavorable response to clinical treatment presented higher levels of NF-κß, ICAM-1 and IL-1ß expression than before treatment^21^. Since NF-κß induces transcription of both IL-1ß and ICAM-1, we hypothesized that NF-κß could be considered a pivotal mediator for the initiation of NP and resistance to GC.

Thus, the aim of this study is to observe whether TNF-α induces the expression of inflammatory recruiters in polyp-derived fibroblasts, and whether fluticasone propionate (FP) inhibits this inflammatory response, in an *in vitro* model. Additionally, the mechanism by which this glucocorticoid acts was evaluated through protein and mRNA levels of VCAM-1, ICAM-1, eotaxin and RANTES, as well as by nuclear translocation of NF-κβ.

## MATERIALS AND METHODS

**Figure 1:**
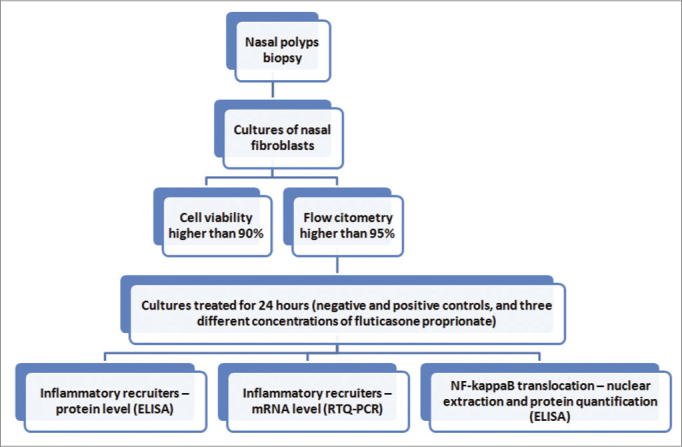
Fluxogram of the study protocol

The study delineation is summarized in Figure 1. Samples from six patients indicated to surgery after clinical treatment failure were studied. For each case bilateral inflammatory NP was confirmed by CT scans and nasal endoscopy. Patients with associated systemic diseases such as ciliary dyskinesia, cystic fibrosis, AERD (aspirin exacerbated respiratory disease) or severe asthma were excluded. Prior to surgery all patients were kept free of any medication for one month. The present study was approved by the local IRB (process number 4374/2007).

### Cell culture

During the surgical procedure, a polyp biopsy was aseptically collected. The polyps were minced into 0.5 mm fragments with surgical knife, and the fragments were then disaggregated with collagenase type IV for 2 hours. Following the enzymatic treatment, the cells were centrifuged and the collagenase solution removed and replaced with culture medium HAM-F10 (supplemented with 1% penicillin, 1% streptomycin, and 20% fetal calf serum), and cultured at 37^o^C in a 5% CO_2_ atmosphere.

After reaching 90% of confluence (one million cells/flask) the cells were trypsinized and the fibroblast component was confirmed by flow cytometry, following the protocol of Saalbach et al.^22^.

Polyp cultures containing at least 90% fibroblasts were then replicated into 5 different flasks and after 24hs treated as follows: negative control (without additive), positive control (TNF-α 25ng/mL), FP1 (TNF-α 25ng/mL and FP 1nM/ 0.45μg/mL), FP10 (TNF-α 25ng/mL and FP 10nM/4.5μg/mL) and FP100 (TNF-α 25 ng/mL and FP at 100nM/45μg/mL). These flasks, as their duplications, were incubated at 37^o^C in a 5% CO_2_ moist atmosphere for another 24 hours.

Treated cells were subsequently trypsinized and stored in Trizol® (for the study of RNAs) and DMSO (for nuclear extraction) at -80^o^C. The culture medium was stored at -20 ^o^C for the ELISA study of the secreted molecules. Cell viability was confirmed to be higher than 90% through Trypan blue exclusion.

### Elisa

Protein concentration of eotaxin, RANTES, sICAM-1 and sVCAM-1 was quantified in the culture medium by ELISA according to the manufacturer instructions (Bio-source, CA, USA).

To normalize the results, the specific protein quantification was corrected according to total protein measured by the method of Bradford, as stated by Protein Assay-Bio Rad dye manufacturer.

### PCR

RNA was extracted with Trizol®, and cDNAs were obtained with the High Capacity cDNA kit. The TaqMan® primers (Applied Biosystems) Hs00164932_m1 (ICAM-1), Hs00237013_m1 (eotaxin), Hs00174575_m1 (RANTES) and Hs00365486_m1 (VCAM-1), in addition to the housekeeping Hs00266705_g1 (GAPDH), were used. The primer concentration was 900 nM and the TaqMan probe concentration was 300nM.

Sample was diluted to 1:10, and 9μL of the dilution was added to 10μL Universal PCR Master Mix (Applied Biosystems) and 1μL of the probe. The 7500 Real-Time PCR System® was employed for analysis (PE Applied Biosystems).

Each sample was tested in duplicate. In all reactions, the same negative controls and calibrators were amplified in parallel to determine the efficiency of all experiments.

The threshold value of 0.1 was determined for gene study. Relative gene expression was quantified using the 2^-ΔΔCT^ method and compared to GAPDH expression in the same sample. The normalized value of each sample was then divided by the calibrator, whose final expression value was assumed to be 1.

### Nuclear extraction

To evaluate the nuclear translocation of NF-kB, the cell membranes were ruptured and the nuclei were isolated as follows: cells were centrifuged at 16000*g* for 5 minutes and washed twice in ice-cold PBS. Then, the pellet was incubated with ice-cold buffer A (10mmol/L HEPES, pH7.9, 10mmol/L KCl, 0.1mmol/L EDTA, 0.1mmol/L EGTA, 1mmol/L DTT, 1mg/L aprotinin, 1mg/L leupeptin, and 1mg/L pepstatin A) for 15 minutes. After cell membrane lysis, 5μL 0.1% NP-40 was added and the solution was vigorously vortexed for 1 minute. The solution was then centrifuged at 20800*g* for 5 minutes at 4^o^C. The supernatant (corresponding to the cytoplasmic fraction) was discarded and the nuclear pellets were suspended in 50μL of ice-cold buffer B (20mmol/L HEPES, pH 7.9, 420mmol/L NaCl, 0.1mmol/L EDTA, 0.1mmol/L EGTA, 1mmol/L PMSF, 1mmol/L DTT, 1mg/L aprotinin, 1mg/L leupeptin, and 1mg/L pepstatin). This new solution was kept at 4°C for 30 minutes with periodic vortexing and centrifuged at 20800*g* for 5 minutes at 4°C. The protein quantification of NF-κß was performed in this final nuclei solution, through ELISA, as described above.

### Statistical analysis

Results were analyzed by the Student t-test for paired samples, with the level of significance set at *p*<0.05.

## RESULTS

### Characterization of fibroblast cultures

After evaluating the viability of cells, a fraction was submitted to flow cytometry in order to confirm that the cultures were specifically composed by fibroblasts. Five of them displayed more than 98% positivity for CD90 and less than 1% positive for CD34, confirming that they were almost exclusively fibroblasts. The other remaining sample presented only 92% of cells positive to CD90 and was excluded from the study.

### Inflammatory recruiters - protein level

**Figure 2:**
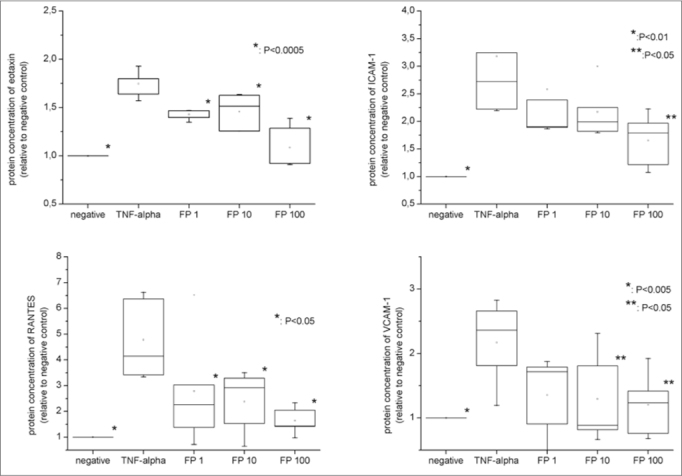
Protein levels of eotaxin, RANTES, VCAM-1 and ICAM-1 measured by ELISA. All the relative values were compared to negative control (considered as 1.0).

When compared to the negative control, TNF-α, at 25ng/mL, significantly increased protein concentrations for ICAM-1 (3.17 fold ±0.77, *p*=0.0075), VCAM-1 (2.17 fold ±0.66, *p*=0.0173), eotaxin (1.74 fold ±0.14, *p*=0.0003) and RANTES (4.78 fold ±1.57, *p*=0.0327) (Figures 2a to 2d). FP decreased the inflammatory recruiters in a dose-dependent manner for all the studied proteins (Figure 2). The reduction was significantly different from the positive control at all FP concentrations for eotaxin (*p*=0.0003) and RANTES (*p*=0.0327), for the 10 nM and 100nM FP treatments for sVCAM-1 (*p*=0.0402) and 100 nM of FP for sICAM-1 (*p*=0.0476).

Additionally, protein concentrations of sVCAM-1 and RANTES were statistically similar to the negative control at all FP concentrations. In contrast, sICAM-1 levels were significantly higher than the negative controls at all concentrations (*p*=0.0175 for FP 100nM) while eotaxin levels were significantly higher than negative control at FP 1 and 10nM concentrations (*p* <0.0001 for 1nM and 0.0057 for 10 nM), but reached similarity at the concentration of 100nM.

### Inflammatory recruiters - mRNA level

The expression of ICAM-1, VCAM-1, RANTES and eotaxin were analyzed though RTQ-PCR. Unfortunately, eotaxin expression was present only when fibroblasts were exposed to TNF-a, which impaired statistical analysis.

**Figure 3:**
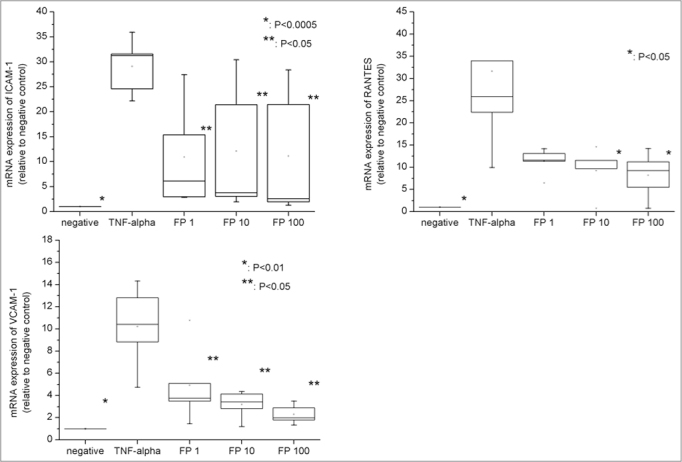
mRNA expression (by RTQ-PCR) of VCAM-1, ICAM-1 and RANTES. All the relative values were compared to negative control (considered to be = 1.0).

TNF-α significantly increased mRNA expression of ICAM-1 (29.09 fold ±5.60, *p*=0.0004), VCAM-1 (10.22 fold ±3.72, *p*=0.0052) and RANTES (31.62 fold ±8.72, *p*=0.0312) (Figure 3). Moreover, RTQ-PCR tests revealed that FP decreased their expression in a progressive manner (Figure 3): the reduction was significantly different from the positive control at all FP concentrations for sVCAM-1 (*p*=0.0488) and for sICAM-1 (*p*=0.0368) and at concentrations higher than 10nM of FP for RANTES (*p*=0.0439).

In addition, the mRNA expression was similar to the negative controls at all FP concentrations for sICAM-1. In contrast, sVCAM-1 and RANTES expressions were significantly higher compared to the negative controls at all the FP concentrations studied.

### Nuclear translocation of NF-κβ

**Figure 4:**
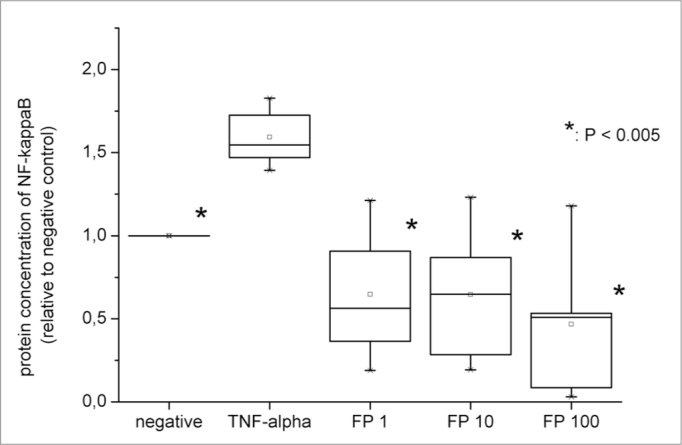
Protein levels of nuclear NF-κß measured by ELISA. All the relative values were compared to negative control (considered as 1.0).

Finally, to investigate a possible relation between NF-κß and increased expression of inflammatory recruiters, we studied whether TNF-α and FP interfered with NF-κß nuclear translocation. For this purpose, cells were submitted to nuclear extraction, and protein concentration of NF-κß was measured in the nuclear fraction. The nuclear levels of this transcriptional factor also increased significantly when fibroblasts were exposed to positive stimulation (1.59 fold ±0.18, *p*=0.0018) (Figure 4).

Also, we assessed if FP reduced both mRNA and protein expression of adhesion molecules and chemokines due to NF-κß inhibition. The ELISA experiment showed that FP significantly decreased NF-κß translocation even at the lowest concentration (1nM) (*p*=0.0022). The levels of nuclear NF-κß were statistically similar to those in the negative control for all FP concentrations (Figure 3).

## DISCUSSION

The fibroblasts of nasal polyps, when induced by a variety of stimuli, can produce pro-inflammatory mediators. Meyer et al.^23^ compared the capacity of TNF-α, IL1-ß or IFN-γ of increasing the expression of RANTES in nasal polyp fibroblasts and observed that TNF-α was a powerful pro-inflammatory stimulus. Based on this study, we chose TNF-α as the positive control for our experiment. We observed that fibroblasts habitually secrete VCAM-1, ICAM-1, eotaxin and RANTES in the culture medium and that TNF-α can significantly intensify the production of these mediators in fibroblasts cultures.

Silvestri et al.^7^ assessed the effect of TNF-α and IL-4 on the expression of ICAM-1 and VCAM-1 by flow cytometry and on the secretion of eotaxin by ELISA in nasal polyp fibroblasts. The authors observed a significant increase of ICAM-1 and eotaxin expression after 24 hours of exposure to progressive concentrations of TNF-α, although VCAM-1 levels remained stable. Similarly, Yoshifuku et al.^24^ observed the effect of TNF-α and IL-4 on the expression of eotaxin, RANTES and VCAM-1 in nasal polyp fibroblast using ELISA and demonstrated that TNF-α at the dose of 10 ng/mL for 24 hours was able to induce the secretion of RANTES and VCAM-1, but not of eotaxin.

Ohori et al.^25^ reported that the expression of VCAM-1 (evaluated by ELISA, flow cytometry and RT-PCR) in nasal fibroblasts was induced by TNF-α in a dose-dependent profile, with a peak at about 10 ng/mL, and reduced at next evaluated dose, 100 ng/mL. TNF-α also induced nuclear translocation and consequently activated NF-κß.

We believe that the differences in expression of some chemokines or adhesion molecules may be related to the use of different concentrations of TNF-α, or to different techniques for the assessment of protein concentration and gene expression. However, there is agreement about the fact that, in general, TNF-α induces the expression of mRNA and the protein production of inflammatory recruiters. The data obtained in the present work are in accordance with this pattern of expression. We also observed that TNF-α significantly increased the nuclear translocation of NFκß, confirming that this is an important mechanism of induction of pro-inflammatory mediators in NP, as stated by Ohori et al.^25^.

Thus, our results, as well as previous reports in literature, agree that the main mechanism in which TNF-α induces cytokines production is through activation of TFs. The TFs, when activated, translocate to the nucleus and interact with cell DNA, increasing the transcription of pro-inflammatory genes.

The mechanism of action of GCs has not been fully elucidated, but its main anti-inflammatory effect is believed to be mediated by the inhibition of TFs 18,^19^.

We observed that FP acts on nasal polyps fibroblasts, reducing the protein secretion and gene expression of inflammatory molecules. This reduction in the protein secretion was significantly different from positive control for RANTES and eotaxin at initial concentrations, and at higher concentrations of FP for ICAM-1. The protein production of VCAM-1 and RANTES remained quite similar to the observed for the negative control at all FP concentrations even though eotaxin and ICAM-1 presented concentrations significantly higher than control levels until treatment with 100nM of FP.

FP also significantly decreased gene expression of ICAM-1 and VCAM-1 at all FP concentrations, while RANTES was significantly decreased after the 10nM treatment. However, the expression of RANTES was still significantly higher compared to negative control until 100nM of FP.

The present results are in agreement with those reported by Silvestri et al.^7^, who observed that FP inhibited eotaxin secretion by the fibroblasts starting at a low dose of 1 nM, whereas it inhibited ICAM-1 only at higher doses (10 nM). Meyer et al.^23^ studied the effect of betamethasone and hydrocortisone on nasal fibroblasts, and demonstrated that these drugs were also effective in inhibiting the RANTES expression.

FP also significantly inhibited the nuclear translocation of NF-κB in fibroblasts at very low FP concentration. This effect on NF-κB in nasal polyp fibroblasts had already been suggested by Silvestri et al.^7^ and by our group^21^, although this is the first *in vitro* study to confirm the effect of FP on NF-κB translocation for nasal polyps.

The mechanism of action of topical GCs on NP should be better recognized in order to improve the efficacy of clinical therapy for nasal polyps. New GCs, that specifically inhibit TFs, or TFs inhibitors, seem to be a reasonable rationale for new therapeutic approaches for this disease.

## CONCLUSIONS

TNF-alpha significantly induced pro-inflammatory mediators production, both at mRNA and protein levels, in nasal polyps fibroblasts cultures.

Also, PF considerably decreased the mRNA and protein expression of all the pro-inflammatory mediators investigated in the present study. This inhibition in inflammatory process was related to a decrease in the nuclear translocation of NF-kappaB.
